# Livestock and Poultry Production in Nepal and Current Status of Vaccine Development

**DOI:** 10.3390/vaccines8020322

**Published:** 2020-06-19

**Authors:** Uddab Poudel, Umesh Dahal, Nabin Upadhyaya, Saroj Chaudhari, Santosh Dhakal

**Affiliations:** 1 Paklihawa Campus, Institute of Agriculture and Animal Science (IAAS), Tribhuvan University, Siddharthanagar-1, Rupandehi 32900, Nepal; poudeluddab15@gmail.com; 2 National Vaccine Production Laboratory, Department of Livestock Services, Kathmandu 44600, Nepal; umeshvet@gmail.com (U.D.); sarojchaudhari@outlook.com (S.C.); 3 Veterinary Standards and Drug Regulatory Laboratory, Budhanilkantha, Kathmandu 44600, Nepal; drnabinvet@gmail.com; 4 W. Harry Feinstone Department of Molecular Microbiology and Immunology, Bloomberg School of Public Health, Johns Hopkins University, Baltimore, MD 21205, USA

**Keywords:** infectious diseases, livestock, poultry, vaccine

## Abstract

The livestock and poultry sectors are an integral part of Nepalese economy and lifestyle. Livestock and poultry populations have continuously been increasing in the last decade in Nepal and are likely to follow that trend as the interests in this field is growing. Infectious diseases such as Foot and Mouth Disease (FMD), Peste des Petits Ruminants (PPR), hemorrhagic septicemia (HS), black quarter (BQ), swine fever, avian influenza, and Newcastle disease (ND) constitute one of the major health challenges to the Nepalese livestock and poultry industry. Vaccinations are an efficient means of preventing the occurrence and spread of several diseases in animals and birds. Considering this fact, the government of Nepal began the production of veterinary vaccines in the 1960s. Nepal is self-reliant in producing several vaccines for cattle and buffaloes, sheep and goats, pigs, and poultry. Despite these efforts, the demand for vaccines is not met, especially in the commercial poultry sector, as Nepal spends billions of rupees in vaccine imports each year. There is a need of strengthening laboratory facilities for the isolation and characterization of field strains of pathogens and capacity building for the production of different types of vaccines using the latest technologies to be self-reliant in veterinary vaccine production in the future in Nepal.

## 1. Status of Livestock and Poultry Population and Productivity in Nepal

According to the Food and Agriculture Organization (FAO), livestock production accounts for 20–24% of the agricultural gross domestic product (AGDP) in developed and developing countries [1]. Globally, more than 600 million households depend on the livestock sector as an essential source of income generation. Livestock contributes to 34% of protein intake and 18% of dietary energy supplies worldwide [1]. Foods from animal sources, including eggs, milk, and meat, can provide high-quality protein and different types of micronutrients, such as vitamin A, riboflavin, calcium, iron, zinc, and vitamin B-12, which cannot be obtained in adequate quantities from plant sources [2]. Moreover, owing to the population growth, the FAO estimates that the demand for livestock-related products will increase by more than 50% by 2050, and it will be mainly driven by the increased demands in Africa and South Asia [1]. These facts highlight that countries should invest in their livestock sector development.

Nepal is an agrarian country where about 66 percent of its population is involved in agricultural occupation [3] Agriculture contributes to around 27.1 percent of the gross domestic product (GDP) of Nepal [4], of which, the livestock sector contributes about 11.5 percent of the total GDP [5] and 25.7 percent of the agricultural GDP (AGDP) [6]. Livestock and poultry sectors contribute to the food and nutritional security by providing meat, milk, and eggs; provide raw materials such as wool and hides for industries; and, also, provide draught power and manure for crop production. In the past decade, the population of cattle, buffalo, goat, pig, chicken, and duck has increased, while that of sheep has declined in Nepal (Table 1) [5]. Nepal produced 1.4 million metric tons of milk in 2017/18, of which the cattle source contributed 36.04% and buffalo contributed 63.96% (Table 2). Milk production has increased by 44.76% in the past decade. Meat production in general has changed by 23.89% in the last 10 years, which was mainly influenced by chicken meat. A tremendous growth was observed in poultry population (Table 1) and production (Table 2) in the past decade in Nepal, which has resulted in the independent contribution of the poultry sector in the national GDP by about 4% [7]. 

The government of Nepal, through different agricultural policies, has acknowledged the importance of its livestock subsector and has contributed towards its development [8]. These agricultural policies, of many things, emphasized the necessity of the genetic improvement of breeds, commercialization of the livestock sector, and promoted public-private partnerships [8]. In recent years, the government of Nepal has prioritized youth-oriented self-employment programs and also started providing livestock insurance and subsidies. Given the cash-generating nature of the livestock sector and increased availability of trainings and infrastructures from the government and nongovernmental sectors, interest in the livestock sector in Nepal is likely to increase in the coming years, resulting in increased livestock population and productivity. 

## 2. Status of Livestock and Poultry Diseases in Nepal

Various bacterial, viral, and parasitic infections cause tremendous economic loss in the livestock and poultry industry globally. Highly pathogenic avian influenza, for example, has already cost billions of dollars to developing countries worldwide [9]. Besides the economic loss in the veterinary sector, several pathogens of animal origin can also spill over into human populations, leading to larger outbreaks [10]. For example, the H1N1 subtype of the influenza A virus, which went through multiple reassortment events in a swine host, was responsible for the 2009 influenza virus pandemic, leading to over 200,000 deaths [11,12].

The World Organization for Animal Health (OIE) has listed several animal diseases, infections, and infestations as globally important and notifiable diseases [13]. There is a high incidence and prevalence of OIE-listed notifiable diseases in livestock and poultry (Table 3) in the rural and urban areas of Nepal, leading to serious threats to animal health and massive economic loss in the livestock industry [14]. During the year 2018, larger outbreaks of Foot and Mouth Disease (FMD), black quarter (BQ), hemorrhagic septicemia (HS), actinomycosis, babesiosis, and theileriosis were recorded by the Veterinary Epidemiology Center (VEC) of Nepal in Cattle and Buffalo (Table 3), with sporadic outbreaks of diseases including anthrax, brucellosis, and tuberculosis (data not shown) [14]. FMD, a highly contagious disease caused by a positive-sense, single-stranded RNA virus of the Picornaviridae family [15]; HS, an acute, fatal, and septicemic disease caused by *Pasteurella multicide* [16]; and BQ, caused by *Clostridium chauvoei* [17], together constitute a major health hazard in the dairy sector of Nepal [18]. Peste des petits ruminants (PPR), caused by *Morbillivirus,* was a major disease outbreak in sheep and goats in Nepal, which is known to result in 1–2 billion dollars losses annually worldwide [19]. Since the poultry industry is growing rapidly, the disease outbreaks are also concurrently observed more in this sector (Table 3). In 2018, over 1000 outbreaks of various viral, bacterial, and protozoal infections were reported in the poultry industry of Nepal, which included coccidiosis, fowl pox, Newcastle disease (ND), highly pathogenic avian influenza, and others (Table 3). 

## 3. Zoonotic Disease Transmission from Livestock and Poultry to Humans in Nepal 

Zoonotic diseases account for more than 60% of the emerging and reemerging diseases in the global population [20]. The burden of zoonotic diseases is massive in low-income countries, like Nepal, where people are highly reliant on livestock production and have limited access to healthcare facilities [21]. The lack of sufficient awareness of Nepali farmers about zoonoses and disease prevention measures highlights the existing risk of pathogen spillover from livestock and poultry species to humans [22,23]. In fact, several bacterial (e.g., brucellosis and salmonellosis), viral (e.g., rabies and avian influenza), and parasitic (e.g., cysticercosis and hydatidosis) zoonoses are endemic in Nepal and are imposing a significant public health burden (Table 4) [23,24]. Rabies, caused by an RNA virus of the genus Lyssavirus from the family *Rhabdoviridae*, is an endemic viral zoonoses in Nepal that results in deaths of around 100 animals and 10-100 humans each year [25]. In addition, about 1000 animals and 35,000 humans receive rabies post-exposure prophylaxis annually [25]. The Ministry of Health and Population (MoHP) of Nepal has recognized six diseases as prioritized zoonoses based on their epidemic potential (Table 4) [26]. The parasitic zoonoses, including neurocysticercosis, toxoplasmosis, and hydatidosis, are estimated to cause a public health impact equivalent to a human immunodeficiency virus infection and acquired immune deficiency syndrome (HIV/AIDS) and higher than that of malaria in Nepal [24]. Serological surveillance for bacterial zoonoses like brucellosis, caused by the Gram-negative bacteria *Brucells* sp., and leptospirosis, caused by spirochetes of the genus *Leptospira*, in humans have indicated the potential spread of these pathogens from animals to humans in Nepal [27]. Periodic outbreaks of the influenza virus have been reported in birds in Nepal since the year 2009 [28]. Recently, a human infection with highly pathogenic avian influenza (H5N1 subtype) has been reported in Nepal, highlighting the risk of the zoonotic transmission of viruses from birds to humans [29]. Vaccines against a few zoonotic diseases, including anthrax, Newcastle disease, rabies, and FMD, are available and are being practiced in Nepal in livestock and birds. However, vaccines against avian influenza and parasitic pathogens are either not available or not in regular practice. 

## 4. History of Livestock and Poultry Vaccination and Vaccine Production in Nepal

The use of vaccines has resulted in the effective management of various livestock and poultry diseases worldwide. Vaccines currently available for veterinary use include inactivated (killed) vaccines, live-attenuated vaccines, toxoids, recombinant subunit vaccines, ribonucleic and deoxyribonucleic acid (RNA/DNA)-based vaccines, and vectored vaccines [34,35]. Vaccine-induced infection control and disease prevention in livestock and poultry are mainly driven by the development of a neutralizing antibody response and pathogen-specific T-cell responses [34,36]. Several novel technologies, including nanoparticles, are being sought for the development of potent vaccines and adjuvants for veterinary use [34,35,37]. 

**Figure 1:**
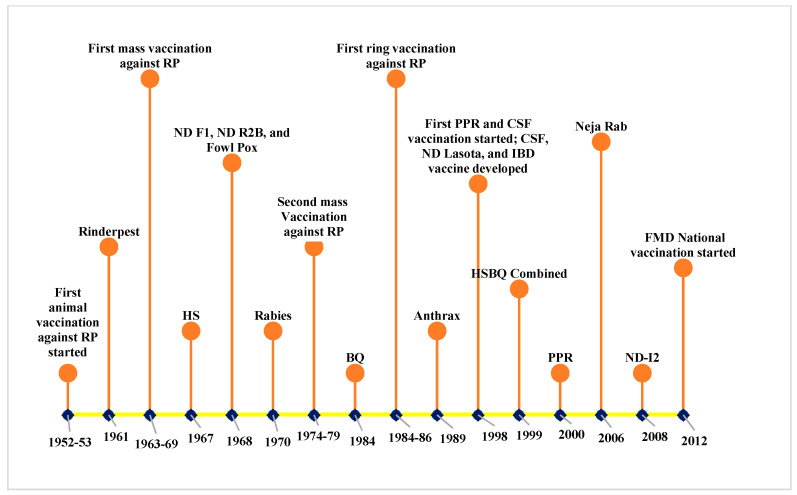
History of vaccination and vaccine production for livestock and poultry in Nepal. Abbreviations: RP—rinderpest, HS—hemorrhagic septicemia, FMD—foot and mouth disease, ND—Newcastle disease, BQ—black quarter, CSF—classical swine fever, PPR—Peste des Petits Ruminants, and IBD—infectious bursal disease.

Nepal has a long history of vaccine production and vaccination (Figure 1). Animal vaccination was practiced first in 1952/53 against rinderpest using the goat tissue vaccine (GTV) imported from India. In 1961, the Veterinary Investigation Laboratory (VIL) was established in Nepal to produce rinderpest GTV [38]. Mass rinderpest vaccinations from 1963–1969 and 1974–1979 immunized over 3 and 4.5 million cattle and buffalo, respectively [39]. The OIE declared that Nepal was rinderpest-free in May 2002. With the enormous efforts from countries all over the world, including Nepal, rinderpest was declared eradicated globally on May 2011 [40]. In 1971, VIL was reorganized as the Central Biological Production Laboratory (CBPL), which subsequently produced different livestock and poultry vaccines. The Rabies Vaccine Production Laboratory (RVPL), established in 1970, switched from the original nerve tissue vaccine technology to cell culture-based vaccine production in 2006 [25]. Vaccinations against FMD at the national level was started in 2012. Getting control over FMD through continuous vaccination is of utmost importance in Nepal, because FMD outbreaks restrict the export of livestock products to other countries [41]. In 2018, the CBPL was renamed the National Vaccine Production Laboratory (NVPL). 

## 5. Current Status of Livestock and Poultry Vaccine Production in Nepal 

At present, there are three laboratories registered for veterinary vaccine productions in Nepal. NVPL is the government-owned laboratory, while Hester Biosciences Nepal and Biovac Nepal are the private organizations for veterinary vaccine productions. NVPL produces at least 14 different types of vaccines for poultry, swine, cattle, buffalo, sheep, goat, and other animals [39]. The quality of vaccines produced by NVPL are within the quality and standards recommended by the OIE, and it is self-reliant in producing vaccines intended to use in the National Livestock Disease Control Program (NLDCP), except for FMD [42]. The vaccines produced by NVPL are available to the farmers mostly free of cost through the NLDCP or at nominal prices recommended by the Nepal government via stockists [39]. The private vaccine production companies are relatively new. They produce vaccines for poultry and large animals, but their quantity is not disclosed publicly. 

The number of vaccines being produced by NVPL is increasing each year (Table 5). In the year 2018/19, NVPL produced around 30 million doses of poultry vaccines, and the ND F1 vaccine alone accounted for half of the these. The production of poultry vaccines was increased at an average rate of 10% to 50% per year in Nepal (Table 5). The HS and BQ combined vaccine, intended for use in cattle and buffalo, was produced in a quantity of around 650,000 doses in the year 2018/19, which was 27% higher than the production five years before. Likewise, the production of the PPR vaccine, which was around eight million doses in 2018/19, was around 166% higher than the production five years before. Similarly, the production of swine fever vaccines to be used in pigs was also increased at a yearly rate of 11.7%, with around 650,000 doses being produced in the year 2018/19 (Table 5). 

There was a massive increase, over 300%, in the import of poultry-related vaccines in Nepal during the last five years (Table 6). Despite this increase in poultry vaccine imports, the import of the ND-I2 vaccine was completely stopped due to enough production of it during recent years by NVPL itself. NVPL has not started production of an FMD vaccine yet in Nepal, and hence, this vaccine needs to be imported. The import of FMD was around 2.4 million doses in the year 2018/19, which is around 90% above the import a half-decade before. A tremendous decline in the import of HS+BQ, HS+BQ+FMD, and swine fever vaccines observed in Nepal in the last five years were attributed to the increased production of these vaccines by the NVPL, which was cheaper and more easily available. 

## 6. Conclusions and Future Perspectives 

The past decade observed a tremendous growth of livestock and poultry productivity in Nepal, and the trend is likely to follow in the future. Hence, the government of Nepal should continue prioritizing agriculture and livestock sectors in their developmental plans and annual budgets. More investments will be necessary to develop breeds that will thrive in the local environment and provide greater returns on investments. Focus should be towards improving the technical knowledge of the farmers, making the feeds and fodder accessible all year round, and improving the diagnostic and therapeutic services. 

Moreover, the number of vaccine doses being produced is increasing over the years. Frequently, vaccine technology is also upgraded, and new vaccines are introduced in the vaccine production pipeline. Despite the commendable contribution of the NVPL in vaccine productions and distributions in Nepal, the demands for livestock and poultry vaccines are highly unmet. This is especially true for the poultry sector. Considering the growth in livestock and poultry production, the demand for vaccines is also going to increase in the future. Hence, the NVPL needs to increase its capacity to produce vaccines against multiple pathogens and in even larger quantities. Introduction of the latest vaccine technologies, expansion of laboratory facilities, and regular training and the capacity building of laboratory personnel would help in the direction of meeting the unmet vaccine demands. Private sectors should also increase their investments for the research and development of vaccines and establish industries with the capability to produce vaccines in large quantities. 

## Authors Contributions 

Conceptualization, U.P. and S.D.; data acquisition U.P., N.U., S.C., U.D., and S.D.; writing—original draft preparation, U.P. and S.D.; writing review and editing, U.P., N.U., S.C., U.D., and S.D.; and supervision U.D. and S.D. All authors have read and agreed to the published version of the manuscript.

## Figures and Tables

**Table 1:** Trend of livestock and poultry populations in Nepal in the last decade (2008/9–2017/18) [5].

Livestock Category	Population in 2008/09 (in Million)	Population in 2017/18 (in Million)	Change in 10 Years (%)	Change/Year (%)
Cattle	7.17	7.37	2.80	0.28
Buffalo	4.68	5.27	12.76	1.28
Sheep	0.80	0.80	−0.27	−0.02
Goat	8.47	11.64	37.42	3.74
Swine	1.04	1.43	37.5	3.75
Chicken	24.48	72.24	195.09	19.50
Duck	0.38	0.40	5.48	0.55

**Table 2:** Milk, meat, and egg productions in Nepal [5].

Product		Production in 2008/9	Production in 2017/18	Change in 10 Years (%)	Change/Year (%)
Meat (MT)	Buffalo	156,627	185,180	18.22	1.82
	Mutton/Chevon	51,183	73,556	43.71	4.37
	Pork	16,992	28,214	66.04	6.60
	Chicken	16,662	60,122	260.83	26.08
	Duck	226	280	23.89	2.38
	Total	241,690	346,179	43.23	4.32
Milk (MT)	Cattle	413,919	754,126	82.19	8.21
	Buffalo	1,031,500	1,338,277	29.74	2.97
	Total	1,445,419	2,092,403	44.76	4.47
Egg (billion)		0.62	1.51	143.54	14.35

**Table 3:** Status of the World Organization of Animal Health (OIE)-listed major disease outbreaks of livestock and poultry in the year 2018 (January–December) [14].

Disease	Species Mainly Affected	No. of Outbreaks	Number of Animals/Birds		
			Susceptible	Affected	Dead
Foot and Mouth Disease (FMD)	Cattle and Buffalo	271	150,669	18,556	311
Black Quarter (BQ)	Cattle and Buffalo	58	41,275	968	42
Hemorrhagic Septicemia (HS)	Cattle and Buffalo	57	6182	2864	92
Actinomycosis/Lumpy Jaw	Cattle and Buffalo	52	122	493	12
Babesiosis	Cattle and Buffalo	28	503	148	11
Theileriosis	Cattle and Buffalo	23	65	255	0
Peste des Petits Ruminants (PPR)	Sheep and Goat	75	232,096	3305	1139
Enterotoxaemia	Sheep and Goat	46	600	1065	25
Classical swine fever	Swine	6	1783	142	39
Coccidiosis	Poultry	583	68,272	168,442	9543
Fowl Pox	Poultry	252	11,332	11,771	766
Infectious Bursal Disease	Poultry	116	183,567	234,848	14,586
Newcastle disease	Poultry	90	901,866	74,986	7363
Highly pathogenic Avian Influenza	Poultry	3	25,254	3110	3110

**Table 4:** Prioritized zoonotic diseases in humans listed by the government of Nepal. GI: gastrointestinal, CNS: central nervous system.

Diseases		Causative Agents	Route of Transmission	Clinical Signs
**Parasitic**	Taeniosis/Cysticercosis/Neurocysticercosis [24]	Taenia spp., especially Taenia solium	Meat-borne and Fecal-oral	GI disorders, severe headache, convulsion, and epileptic seizures
	Hydatidosis [30]	Echinococcus granulosus	Fecal-oral	Cyst in liver, CNS, and lungs, leading to coughing, chest pain, and breathing difficulty
	Toxoplasmosis [31]	Toxoplasma gondii	Meat-borne and Fecal-oral	abortion, still-birth, congenital abnormalities, and vision impairment
**Bacterial**	Leptospirosis [32]	Leptospira interrogans	Contact with contaminated soil and water or exposure to animal reservoir	high fever, headache, chills, muscle aches, and jaundice and anemia
	Brucellosis	Brucella abortus	Contaminated food material	Body-ache, night sweats, Malta or Mediterranean fever, poor appetite, weight loss
**Viral**	Avian Influenza [33]	Influenza A virus (H5 and H7)	Direct and indirect contact	Fever, Cough, sore throat, muscle aches, and conjunctivitis

**Table 5:** Trend of livestock and poultry vaccine productions at the National Vaccine Production Laboratory (NVPL), Nepal [43].

Vaccine	Species Used	Dose Produced (×1000)		Change in Last 5 Years (%)	Change per Year (%)
		2014/15	2018/19		
ND F1	Poultry	10,000	15,000.30	50.00%	10.01%
ND Lasota + ND R2B	Poultry	2500	5008.50	100.34%	20.07%
ND − I2	Poultry	576	1963.80	240.93%	48.18%
IBD + Pox	Poultry	5088	8003.70	57.30%	11.46%
HS + BQ	Cattle and buffalo	511	650	27.20%	5.44%
PPR	Sheep and Goat	3000	8000.90	166.67%	33.33%
Rabies	Canine	15.69	120	664.81%	132.96%
Swine Fever	Swine	410	650	58.53%	11.70%

**Table 6:** Trend of livestock and poultry vaccine imports in Nepal [44].

Vaccine	Species Used	Dose Imported (×1000)		Change in Last 5 Years (%)	Change per Year (%)
		2014/15	2018/19		
Poultry vaccines (combined)	Poultry	549,567	2,378,685.85	332.82%	66.56%
FMD	Cattle and buffaloes	1269	2409.43	89.86%	17.97%
HS + BQ	Cattle and buffaloes	4534.20	585.12	−87.09%	−17.41%
HS + BQ + FMD	Cattle and buffaloes	270	0	−100%	−20%
Rabies	Canine	305	297.91	−2.32%	−0.46%
Swine fever	Pigs	200	0.10	−99.95%	−19.99%
